# Big telescope, big data: towards exascale with the Square Kilometre Array

**DOI:** 10.1098/rsta.2019.0060

**Published:** 2020-01-20

**Authors:** A. M. M. Scaife

**Affiliations:** Jodrell Bank Centre for Astrophysics, Department of Physics and Astronomy, University of Manchester, Oxford Road, Manchester M13 9PL, UK

**Keywords:** radio astronomy, computing, image processing

## Abstract

Unlike optical telescopes, radio interferometers do not image the sky directly but require specialized image formation algorithms. For the Square Kilometre Array (SKA), the computational requirements of this image formation are extremely demanding due to the huge data rates produced by the telescope. This processing will be performed by the SKA Science Data Processor facilities and a network of SKA Regional Centres, which must not only deal with SKA-scale data volumes but also with stringent science-driven image fidelity requirements.

This article is part of a discussion meeting issue ‘Numerical algorithms for high-performance computational science’.

## 1. The Square Kilometre Array

The Square Kilometre Array (SKA) telescope will be the world’s largest radio observatory. Comprising two separate telescopes in its first phase, it will cover the radio spectrum from the metre to centimetre-wavelengths. The SKA1-LOW telescope will be constructed in Western Australia at the Murchison Radio Observatory and cover the frequency range 50–350 MHz. Designed primarily to image the Epoch of Reionization, the period in cosmic history when the first stars formed, it will be formed of stations of phased dipole antennas separated by up to 65 km. The SKA1-MID telescope will be constructed in the Karoo Astronomy Reserve of South Africa and will be able to support radio receivers that work up to a frequency of approximately 20 GHz. SKA1-MID will have 197 dish antennas spread over distances of more than 100 km and is designed to address a range of key science areas including the reconstruction of gravitational waves using high-precision pulsar timing, constraining the origin of cosmic magnetic fields and looking for tracers of life from other planets. This paper gives an overview of the data flow for the SKA, describes the major processing steps required for image-based science and highlights some of the specific imaging challenges that face the observatory.

## 2. The SKA data flow

Data from the individual antennas of the SKA1-MID, or the individual stations of the SKA1-LOW, are transported to the central signal processing facility, where the data from each pair of antennas/stations are correlated to produce the visibility data described in detail in §3. For most existing radio telescopes, it is these data that are distributed to the wider astronomy community; however, for the SKA, the rate that these data are produced is approximately 0.5–1 TB s^−1^. Given that a typical SKA observation will be approximately 6 h in length, this results in a volume of data too large for the average astronomer to handle and consequently the subsequent calibration and imaging for the SKA will be performed within the observatory itself in a dedicated processing facility referred to as the Science Data Processor (SDP). For the majority of science cases, the standard observatory data product delivered by the SDP to the international astronomy community will be an image cube, where the two spatial dimensions are accompanied by a spectral frequency axis. Typical images will have spatial axes with 2^15^ pixels on a side and up to 2^16^ frequency channels, although more often this frequency axis will be compressed to three Taylor term coefficients which can concisely express the continuum frequency behaviour of astrophysical radio emission mechanisms. This dimensionality results in petabyte scale volumes for individual data products, and when the data products required for the SKA high-priority science objectives are considered on average they are expected to result in outputs equivalent to approximately 300 PB per telescope per year during full science operations, resulting in an archive of approximately 8.5 Exabytes over the 15-year lifespan of the initial high-priority science programmes [1]. This volume of data is considered to be prohibitive for the average astronomer, or astronomy collaboration, and so it is intended that SKA observatory data products will be delivered to a network of SKA Regional Centres which will provide processing and storage support for the astronomy community.

## 3. Interferometric image formation

Unlike optical telescopes, radio interferometers do not image the sky directly. Instead they measure the amount of power on different angular scales, and these measurements are then used to reconstruct an image of the radio sky. The angular scales that are sampled by a particular interferometer are defined by the pairwise vector separations of the antennas in that array. Each antenna pair is known as a *baseline* and the measurement from a baseline is known as a *visibility*, which is defined as the mutual coherence of the incident electric fields at the two antennas

3.1 $$V_{ij}={\left\langle E(r_{i},t)E^{*}(r_{j},t)\right\rangle }_{t},$$

where *i* and *j* represent the indices of the antennas, 〈〉_*t*_ represents the time average and E* indicates the complex conjugate of the electric field, *E*.

The van Cittert–Zernike theorem shows that *V*_*ij*_ is effectively equivalent to being a Fourier component of the radio sky brightness, *I*(ℓ, *m*), such that

3.2 $$V(u,v,w)=\int \frac{I(\ell ,m)}{\sqrt{1-\ell^{2}-m^{2}}}{\text{e}}^{2\pi j(u\ell +vm+w\sqrt{1-\ell^{2}-m^{2}}-1)}\;\text{d}\ell \text{d}m,$$

where *j* now represents $\sqrt{-1}$, ℓ and *m* are direction cosines and (*u*, *v*, *w*) represent the separation of the antenna pair in a Cartesian frame projected towards the observing direction in units of wavelength. For many interferometers the antenna separation in the forward direction, *w*, is small enough to be neglected and the relationship is approximated as a two-dimensional transform. As the Earth rotates during an observation the projected length of an individual baseline towards a particular observing direction on the sky changes deterministically, increasing the range of Fourier scales that are sampled by a finite array. The directed use of the Earth’s rotation for filling the Fourier plane is known as *Earth rotation aperture synthesis* and was the subject of the 1974 Nobel Prize in Physics [2].

Image formation for radio interferometers inverts this relationship to recover a measured sky brightness distribution. This measured distribution is reconstructed from an incomplete spectrum of Fourier components, sampled at the finite number of antenna separations in the array, *S*(*u*, *v*)

3.3 $$I_{\text{meas}}(\ell ,m)=\int S(u,v)V(u,v){\text{e}}^{2\pi j(u\ell +vm)}\;\text{d}u\text{d}v,$$

which can be rewritten as a sum since

3.4 $$S(u,v)=\sum_{i=1}^{M}\delta (u-u_{i},v-v_{i}),$$

where *M* = 0.5 × *N*_ant_(*N*_ant_ − 1) × *N*_*τ*_ × *N*_*f*_ is the total number of visibility samples across all baselines in an array with *N*_ant_ antennas, for *N*_*τ*_ time samples and *N*_*f*_ spectral channels.

In practice, the formation of *I*_meas_(ℓ, *m*) is done using a fast Fourier transform (FFT) due to the better algorithmic scaling of O(nlog⁡n) compared to the $O(n^{2})$ scaling of the traditional discrete Fourier transform [3]. However, this leads to a complication in the image formation process because an FFT requires data to be regularly sampled in the spatial dimension, but visibility data are instead regularly sampled in time and frequency. To create a regularly spaced grid in Fourier space suitable for use with an FFT, the natively sampled visibilities (known as *continuous* visibilities) are gridded onto a regular array of side $\sqrt{k}$ using a convolutional kernel, *C*_aa_(*u*, *v*), to minimize the effects of aliasing. The resulting grid of measurement data, *V*_grid_(*u*_*k*_, *v*_*k*_), can be expressed mathematically as

3.5 $$V_{\text{grid}}(u_{k},v_{k})=[[V(u,v)\cdot S(u,v)]*C_{\text{aa}}(u,v)]\cdot Ш(u_{k},v_{k}),$$

where $Ш(u_{k},v_{k})$ is the Shah function. The most commonly used convolution kernel in radio astronomy imaging is the prolate spheroidal wave function.

At this point in the imaging process, it is also possible to up-weight or down-weight different areas of the visibility sampling to highlight different spatial features. For example, if sufficient signal to noise is present in the data, it can be sacrificed to up-weight the visibility data from long baselines and improve the resolution in the resulting image. Weighting, *W*(*u*, *v*), is normally applied directly to the continuous visibility data, although this can require multiple passes through the gridding procedure for particular forms of weights to be applied

3.6 $$V_{\text{grid}}(u_{k},v_{k})=[[V(u,v)\cdot S(u,v)\cdot W(u,v)]*C_{\text{aa}}(u,v)]\cdot Ш(u_{k},v_{k}).$$

At this point, *V*_grid_(*u*_*k*_, *v*_*k*_) can be transformed to create an image. The output must be corrected for the convolutional gridding kernel that has been introduced and the weights must be normalized

3.7 $$I_{\text{meas}}(\ell ,m)=\frac{{\text{FT}}^{-1}[V_{\text{grid}}(u,v)]}{\left(\sum W(u,v)\right){\text{FT}}^{-1}[C_{\text{aa}}(u,v)]},$$

where FT^−1^ indicates the inverse Fourier transform. The resulting image is known as the *dirty image* since it has not yet been corrected for *S*(*u*, *v*)*W*(*u*, *v*). This multiplication in Fourier space results in a convolution of the image

3.8 $$V(u,v)\cdot [S(u,v)\cdot W(u,v)]\Leftrightarrow I(\ell ,m)*b_{\text{PSF}}(\ell ,m),$$

where the point spread function, *b*_PSF_(ℓ, *m*), also known as the *synthesized beam* is defined as

3.9 $$b_{\text{PSF}}(\ell ,m)={\text{FT}}^{-1}[S(u,v)\cdot W(u,v)].$$

## 4. Imaging challenges

As described above, the principles of image formation for radio interferometry are well defined at this point. However, there are a number of complications that the telescope faces in terms of exact implementation. These stem largely from the stringent image fidelity requirements imposed by the scientific goals of the SKA and, although algorithmic solutions do exist, the current challenge lies in coping with the resultant processing load required to implement them in light of the large data volumes associated with the SKA. Here, I outline some of the most important elements.

### (a) Undersampling and deconvolution

Since the shape of the synthesized beam, *b*_PSF_(ℓ, *m*), depends on the sampling distribution, *S*(*u*, *v*), it can be improved by adding in additional antennas to the array, or observing for longer periods to take advantage of the Earth’s rotation to fill the Fourier plane; however, regardless of these augmentation techniques a radio interferometer array will always have a maximum antenna separation and a minimum antenna separation. The first of these imposes a finite resolution on the resulting image, the second means that the zero-spacing in the Fourier plane is never measured by an interferometer. These fundamental limitations result in a sampling function that will necessarily be zero-valued across specific areas of the visibility grid, *V*_grid_(*u*_*k*_, *v*_*k*_), and therefore cannot be directly compensated for using division in Fourier space.

The deconvolution process for radio imaging therefore typically takes place in the image plane, using a variant of matching or basis pursuit known as the CLEAN algorithm. This is an iterative deconvolution process which can be implemented in a variety of ways, the most popular of which is known as Cotton-Schwab CLEAN, which subtracts collections of image components directly from the continuous visibility data before repeating the entire imaging process to find the next collection of components [4].

### (b) Spectral behaviour

The synthesized beam can also be improved by filling the Fourier plane using multi-frequency measurements. Modern radio interferometers operate broad-band receivers that recover a highly channelized wide bandwidth measurement. As the length of individual baselines is measured in units of wavelengths this could, in principle, lead to an improved radial filling of the Fourier plane; however, the continuum radio emission mechanisms operating in astrophysical sources have frequency-dependent behaviour that makes this untenable. Directly gridding visibility data from multiple spectral channels to form a single image can lead to significant image plane aberrations. To address this, radio imaging algorithms re-parameterize this spectral behaviour by taking a Taylor expansion of the frequency dependence and imaging (typically) the first three Taylor term coefficients separately [5].

### (c) Non-coplanarity

Although in equation (3.3) we used a planar approximation to simplify the response of the interferometer, this approach is only valid for small field-of-view and/or compactly distributed telescope arrays. In reality, the antennas are distributed in three dimensions and the sky is curved. These considerations are contained in the *w*-parameter of equation (3.2) and neglecting it in the case of wide fields-of-view or non-compact arrays can again lead to image plane aberrations. The effect of the *w*-term in equation (3.2) is to introduce a direction-dependent phase screen that is different for each baseline—effectively, each antenna pair sees a different sky.

Introducing a third dimension to the visibility equation would require a three-dimensional Fourier transform to recover an image. Since such transforms are computationally expensive, especially given that the *w*-axis would be very sparsely sampled, a number of solutions to approach this problem from an alternative direction have been developed. These largely form variations on the original solution, known as *w*-projection [6], where the multiplication of the exponent in the image plane is reformulated as a convolution in Fourier space

4.1 $$V(u,v,w)=V(u,v,w=0)*{\tilde{C}}_{w}(u,v),$$

where ${\tilde{C}}_{w}(u,v)$ is the Fourier transform of the direction-dependent phase term

4.2 $$C_{w}(\ell ,m)={\text{e}}^{-2\pi jw\sqrt{1-\ell^{2}-m^{2}}}.$$

### (d) Direction-dependent calibration effects

Wide-field imaging suffers not only from the *w*-effect but also from a number of other calibration issues that impact the imaging process. Key among these is the effect of the antenna illumination pattern, known as the *primary beam*. Owing to the obscuring effects of the receiver system, and in many telescopes the support structures that hold the receiver, this illumination pattern is asymmetric at large distances from the field centre. This asymmetry becomes problematic for longer observations as the illumination pattern rotates relative to the sky over time, causing a time-dependent gain that varies radially across the field and prevents the deconvolution process from converging.

Much like the *w*-effect, this challenge can be tackled by approaching the image-plane multiplication of the illumination pattern as a convolution in Fourier space. Known as **a*-projection* [7], this process depends on the aperture illumination pattern being precisely known. If this is not the case, then the imaging process must be performed in an interleaved fashion with a self-calibration cycle that determines the direction-dependent amplitude of the primary beam.

## 5. Conclusion

Even without considering the added complexities in the calibration and imaging process that are described in the previous section, the Fourier nature of the measurements in radio interferometry makes SKA data processing computationally challenging. Performing multiple Fourier transforms on data with image sizes as large as 2^15^ or even 2^16^ pixels on a side is computationally prohibitive. Furthermore, the refinements to the basic imaging process such as iterative direction-dependent calibration, deconvolution and convolutional gridding not only increase the number of Fourier transforms required to create science ready data products, but also add in additional processing. In spite of these challenges, the computing for the SKA over the coming decade is certainly achievable, and it is likely that its implementation will be instrumental in driving the next generation of global e-infrastructure.

More details on the design of the data transport and processing systems for the SKA can be found at www.skatelescope.org.

## Data accessibility

This article has no additional data.
